# Comparing antibiotic consumption between two European countries: are packages an adequate surrogate for prescriptions?

**DOI:** 10.2807/1560-7917.ES.2017.22.46.17-00352

**Published:** 2017-11-16

**Authors:** Laurence Watier, Philippe Cavalié, Bruno Coignard, Christian Brun-Buisson

**Affiliations:** 1 Biostatistics, Biomathematics, Pharmacoepidemiology and Infectious Diseases (B2PHI), Inserm, UVSQ, Institut Pasteur, Université Paris-Saclay, Paris, France; 2 Agence Nationale de Sécurité du Médicament (ANSM), Surveillance Division, Saint Denis, France; 3 Santé Publique France, Saint-Maurice, France; 4 Ministère de la Santé, Paris, France

**Keywords:** antibiotic use, ambulatory care, surveillance, indicators

## Abstract

Defined daily doses (DDD) are the gold standard indicator for quantifying prescriptions. Since 2014, the European Centre for Disease Prevention and Control (ECDC) has also been using the number of packages per 1,000 inhabitants per day (ipd), as a surrogate for prescriptions, to report antibiotic consumption in the community and to perform comparisons between European Union (EU) countries participating in the European Surveillance of Antimicrobial Consumption Network (ESAC-Net). In 2015, consumption was reported to range across Europe from 1.0 to 4.7 packages per 1,000 ipd. Our analysis showed that consumption of antibiotics for systemic use per 1,000 ipd was on average 1.3 times greater in France than in Belgium when considering prescriptions in the numerator, 2.5 times greater when considering packages and 1.2 times greater when considering DDD. As long as the same metrics are used over time, antibiotic consumption data aggregated and disseminated by ECDC are useful for assessing temporal trends at the European level and within individual countries; these data may also be used for benchmarking across EU countries. While DDD - although imperfect - are the most widely accepted metric for this purpose, antibiotic packages do not appear suitable for comparisons between countries and may be misleading.

## Background

In order to compute the overall exposure of the population to antimicrobial drugs and compare drug consumption within Europe, countries participating in the European Surveillance of Antimicrobial Consumption Network (ESAC-Net) report each year to the European Centre for Disease Prevention and Control (ECDC) the number of defined daily doses (DDD). The DDD is a widely used metric defined by the World Health Organization (WHO) [1], used for all active substances according to the anatomical therapeutic chemical (ATC) classification. These metrics are mainly based on sales or, to a lesser extent, on reimbursement data for active substances. The data include antibacterial drugs for systemic use (J01), antimycotic drugs for systemic use (J02), antibiotic drugs used for treatment of tuberculosis (J04B) and antiviral drugs for systemic use (J05). The ratio of DDD per 1,000 inhabitants and per day (ipd) is the most widely accepted indicator for comparing antibiotic consumption between countries. The ECDC has been using this indicator for reporting and comparing antibiotic consumptions within and between European Union (EU) countries participating in ESAC-Net (formerly ESAC) since 2001, with retrospective data going back to 1997.

Since 2014, summaries disseminated by the ECDC have also reported the number of orally administered packages (OAP) per 1,000 ipd in the community for the substances classified as antibacterial drugs for systemic use (J01). This additional indicator is described as *“the best available surrogate for prescriptions*” when the latter are unavailable [2-4] and its purpose is to help understand changes in antibiotic consumption [5]. Antibiotic prescriptions are obtained from individual reimbursement data for ambulatory care recorded in national health insurance (NHI) databases [6,7]. However, the validity of this indicator is mainly related to the NHI coverage rate and also affected by the possibility to purchase antibiotics without a prescription. Thus, a surrogate for prescriptions derived from sales data (e.g. packages sold) could be useful. ESAC-Net summaries use OAP per 1,000 ipd to compare EU countries; however, this results in unexpected marked differences depending on whether the ranking of countries is based on DDD or OAP, although the reader is warned that such differences could “*probably reflect differences in the number of items or dose per item of antibiotics in antibiotic packages*” [3,4].

To investigate the validity of OAP, we first compared the two indicators in France during the period from 2006 to 2015. Secondly, since prescription data were publicly available only from Belgium among the countries participating into ESAC-Net [8], reasons for discrepancies in the results of ranking schemes using DDD and OAP in France and in Belgium were investigated for the period from 2006 to 2009.

## Comparison of defined daily doses, prescriptions and packages of antibiotic drugs delivered in France, 2006 to 2015

We used anonymous individual data from the main general scheme of the French NHI agency which insures salaried workers and covered a constant proportion of around 86% of the French population during the study period. These data comprised all prescriptions and antibiotics prescribed to outpatients, dispensed by outpatient pharmacies, and reimbursed by the NHI from 2006 to 2015 (Commission nationale informatique et libertés (CNIL) approval DE-2015–190). To take into account French population growth during the study period, we obtained demographic data from the French National Institute for Statistics and Economic Studies [9]. Prescription data recorded in number of OAP were converted into number of DDD, according to the official DDD published by the WHO for each substance and using the rules established by the WHO to determine the appropriate DDD for combined drugs formulations. The annual number of prescriptions, OAP and DDD prescribed in France per 1,000 ipd were aggregated at the active substance level, i.e. the fifth level. The contents of oral formulations were obtained from the French public database of medicines [10]. All data were extrapolated to 100% of the French population, assuming that the 14% population not covered by the data collection had an antibiotic consumption similar to the 86% covered.

The study targeted oral antibiotics for systemic use (ATC code J01) in the community only. As in ESAC-Net summaries, antibiotics were divided in six main categories according to the ATC level 3 classification: penicillins (J01C), cephalosporins and other beta-lactams (J01D), macrolides, lincosamides and streptogramins (J01F), tetracyclines (J01A), quinolones (J01M) and sulfonamides and trimethoprim (J01E). The other classes accounted for a very small part of overall antibiotic consumption in the community and were thus omitted from the analysis.

## Outpatient antibiotic use in France, 2006 to 2015

Over the 10-year study period, an average of 2.71 (range: 2.55–2.87) prescriptions, 5.10 (range: 4.91–5.33) OAP and 28.94 (range: 26.33–30.22) DDD per 1,000 ipd were recorded for oral antibiotics for systemic use, indicating that ca 1.9 OAP were delivered for each prescription. The number of prescriptions, OAP and DDD per 1,000 ipd varied by ATC class (Figure). The temporal trends using OAP or DDD were quite similar, except for tetracyclines for which a noticeable decrease in OAP was observed because the package size increased during the study period, with ca 1.6 and 1.1 OAP delivered for each prescription of tetracyclines in 2006 and 2015, respectively. For penicillins, the temporal trends of OAP and DDD were similar but increased more than the prescriptions.

**Figure:**
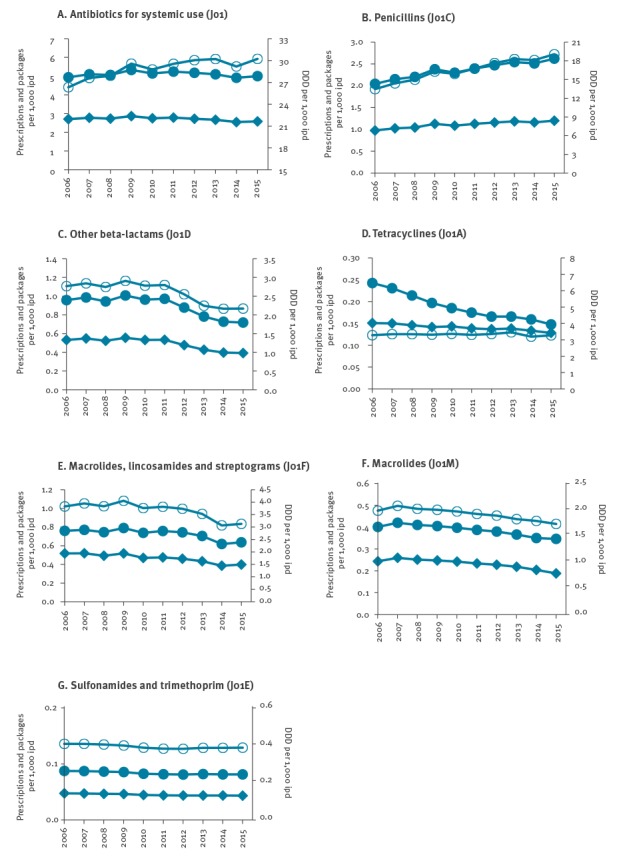
Outpatient antibiotic use per year expressed as number of orally administered packages, prescriptions and defined daily doses per 1,000 inhabitants per day, France, 2006–2015 (n = 535,696,881 prescriptions)

DDD: defined daily doses; ipd: inhabitants per day.

Circles: administered packages; diamonds: prescriptions; blank circles: DDD.

## Comparison of France and Belgium, 2006 to 2009

We compared French data to those recorded in Belgium, the only other EU country from which corresponding data were publicly available for the period from 2006 to 2009. These data were retrieved from the supplementary material of the paper by Coenen et al. [8]. Three 12-month periods from July 2006 to June 2009 could thus be compared. The contents of oral formulations were obtained from the Belgian Centre for Pharmacotherapeutic Information (CBIP) [11].

Over the 3-year study period, the numbers of packages and prescriptions in Belgium were comparable for all ATC classes considered (Table 1). In contrast, the number of packages in France was about twice as high as the number of prescriptions for ATC J01, with the packages: prescriptions ratio varying from 1.5 to 2.2 depending on the ATC class.

**Table 1:** Number of defined daily doses, packages (all administered) and outpatient prescriptions per 1,000 inhabitants and per day, Belgium (n =24,690,829 prescriptions) and France n=161,107,123), 2006–2009

Period	Defined daily doses			Packages			Prescriptions		
	2006–07	2007–08	2008–09	2006–07	2007–08	2008–09	2006–07	2007–08	2008–09
**Belgium**									
J01	23.33	24.85	24.52	2.25	2.30	2.24	2.11	2.16	2.10
J01A	1.82	1.95	1.91	0.13	0.13	0.13	0.12	0.12	0.12
J01C	12.22	13.70	13.65	1.08	1.15	1.12	1.02	1.09	1.07
J01D	2.30	2.07	1.70	0.20	0.16	0.13	0.19	0.15	0.12
J01E	0.34	0.36	0.33	0.05	0.05	0.04	0.04	0.05	0.04
J01F	2.39	2.51	2.54	0.34	0.35	0.34	0.32	0.33	0.32
J01M	2.19	2.10	2.23	0.27	0.26	0.27	0.26	0.25	0.26
**France**									
J01	26.83	27.74	28.70	5.44	5.54	5.66	2.72	2.75	2.80
J01A	3.34	3.34	3.32	0.24	0.22	0.20	0.15	0.15	0.14
J01C	13.81	14.68	15.61	2.12	2.21	2.33	0.99	1.03	1.09
J01D	2.78	2.78	2.83	1.15	1.16	1.18	0.54	0.53	0.54
J01E	0.41	0.40	0.40	0.09	0.09	0.09	0.05	0.05	0.05
J01F	3.84	3.90	3.94	0.76	0.76	0.77	0.51	0.51	0.50
J01M	2.06	2.04	2.01	0.42	0.41	0.41	0.26	0.26	0.25

Compared with Belgium, consumption of antibiotics for systemic use (J01) per 1,000 ipd was on average greater in France for the three indicators: 1.3 times greater when considering prescriptions in the numerator, 2.5 times greater when considering packages and 1.2 times greater when considering DDD.

Variations were also observed according to ATC class. For penicillins, the number of prescriptions and DDD (measured per 1,000 ipd) were similar in France and Belgium, while the number of packages was twice greater in France than in Belgium. Accordingly, the quantity of active substance per package was about half in the French packages compared with Belgium. Table 2 provides examples of package contents for various amoxicillin formulations used in Belgium and in France and confirms that packages available in Belgium contain more active substance. Regarding the trends for penicillins (J01C) use in France, the discrepancy between amoxicillin prescriptions and DDD could be partly explained by the low DDD ascribed to this drug (1 g), which is much lower than daily doses of amoxicillin recommended in French and Belgian guidelines for lower respiratory tract infections in adults (3 g/day) [12,13].

**Table 2:** Content of oral amoxicillin formulations available in Belgium and in France

Brand name	Dosage	Belgium		France	
		Quantity	Active substance	Quantity	Active substance
**Brand A**					
Capsule	500 mg	16 or 30	8 or 15 g	12	6 g
Powder for oral suspension	250 mg/ 5 mL	100 mL	5 g	60 mL	3 g
Powder for oral suspension	500 mg/ 5 mL	100 mL	10 g	60 mL	6 g
**Brand B**					
Capsule	500 mg	16	8 g	12	6 g
Dispersible tablet	1 g	8 or 20 or 24	8 or 20 or 24 g	6 or 14	6 or 14 g
Powder for oral suspension	250 mg/ 5 mL	100 mL	5 g	60 mL	3 g
Powder for oral suspension	500 mg/ 5 mL	100 mL	10 g	60 mL	6 g
**Brand C**					
Capsule	500 mg	16	8 g	12	6 g
Dispersible tablet	1 g	8 or 24	8 or 24 g	6 or 14	6 or 14 g
Powder for oral suspension	125 mg/ 5 mL	80 mL	2 g	60 mL	1.5 g
Powder for oral suspension	250 mg/ 5 mL	80 mL	4 g	60 mL	3 g

Formulations in France and Belgium as per [10,11]. Prescribed daily doses (PDD) could not be assessed from available Belgian data.

## Discussion 

Our analysis comparing trends in the consumption of antibiotic packages and of DDD in France shows that using antibiotic packages as a metric could be misleading. In addition, comparing antibiotic consumption across EU countries using aggregated packages may also lead to erroneous conclusions in terms of antibiotic exposure of the population, unless package contents are similar.

As long as the same metrics are used over time, antibiotic consumption data aggregated and disseminated by ECDC are useful for assessing temporal trends at the European level and within individual countries; these data may also be used for benchmarking across EU countries. While DDD - although imperfect - are the most widely accepted metric for this purpose, antibiotic packages do not appear suitable for comparisons between countries and may be misleading [14,15]. Indeed, while comparable results were found in France and Belgium when assessing antibiotic consumption based on prescriptions or DDD, using packages would have implied that consumption in France was twice as high as in Belgium. Likewise, Slovenia and Sweden had similar consumption if expressed in DDD per 1,000 ipd, but a 42% difference when using OAP [14].

A dialogue should be initiated at the European level with manufacturers and regulatory authorities regarding the size and harmonisation of antibiotic packages. It is conventionally recognised that the drug dosage per package should correspond to the most common indication for adults. In that case, the average number of packages delivered per prescription should be close to 1, as is the case in Belgium, where packages appear to be a good surrogate for prescriptions [8,16,17]. This is not the case in France or Slovenia [14], where packages often contain less active substance than in Belgium or Sweden. The result is that the international comparisons are distorted when packages are taken as a surrogate for prescriptions and antibiotic use. Since there may be different dosages and duration of therapy prescribed for different indications, it is unrealistic to expect that packages accurately reflect prescriptions, unless this is adjusted for prescribed daily doses. Using packages as a metric for exposure to antibiotics may however be useful for examining trends in consumption in specific subgroups. For example, since the daily dose ascribed to each active substance is based on adult dosages, antibiotic consumption in children is underestimated. For that specific age group, examining antibiotic consumption of paediatric packages could be of interest.

Monitoring antibiotic use is a global priority to inform public health policies. Recently, the Drive-AB group proposed a set of indicators, especially for the outpatient setting [18], including, besides DDD, prescriptions and treatment courses per defined population. The US Centers for Diseases Control and Prevention advocate using prescriptions [19]. Development of valid indicators for outpatient antibiotic use allowing country comparisons across the EU is a real challenge [20]. Currently, antibiotic consumption indicators in EU countries are mostly derived from sales data. Because they can be linked to prescriptions and patients, antibiotic consumption indicators using reimbursement data should be developed and used whenever possible. However, this may not be suitable for all EU countries; for example in Spain, some antibiotics are not recorded in the reimbursement database, resulting in underestimation of the actual consumption [6].

## Conclusions

Currently, the use of packages in order to measure the antibiotic consumption appears unsuitable. It could mislead the public to believe that all packages are equal, irrespective of content (dose, number of tablets, etc.). Unless harmonisation of packages is achieved across EU countries, using the number of packages as a measure of antibiotic exposure should therefore be limited to providing additional perspectives in order to explain specific evolutions.

In conclusion, we advocate not using OAP as a surrogate for prescriptions for benchmarking antibiotic consumption across Europe until package content or prescribed daily doses have been harmonised.
